# Numerical Approach to Facial Palsy Using a Novel Registration Method with 3D Facial Landmark

**DOI:** 10.3390/s22176636

**Published:** 2022-09-02

**Authors:** Junsik Kim, Hyungwha Jeong, Jeongmok Cho, Changsik Pak, Tae Suk Oh, Joon Pio Hong, Soonchul Kwon, Jisang Yoo

**Affiliations:** 1 Department of Electronic Engineering, Kwangwoon University, Seoul 01897, Korea; 2 Department of Plastic Surgery, Asan Medical Center, University of Ulsan College of Medicine, Seoul 05505, Korea; 3 Graduate School of Smart Convergence, Kwangwoon University, Seoul 01897, Korea

**Keywords:** 3D facial landmark, facial palsy, iterative closest point, registration, symmetry

## Abstract

Treatment of facial palsy is essential because neglecting this disorder can lead to serious sequelae and further damage. For an objective evaluation and consistent rehabilitation training program of facial palsy patients, a clinician’s evaluation must be simultaneously performed alongside quantitative evaluation. Recent research has evaluated facial palsy using 68 facial landmarks as features. However, facial palsy has numerous features, whereas existing studies use relatively few landmarks; moreover, they do not confirm the degree of improvement in the patient. In addition, as the face of a normal person is not perfectly symmetrical, it must be compared with previous images taken at a different time. Therefore, we introduce three methods to numerically approach measuring the degree of facial palsy after extracting 478 3D facial landmarks from 2D RGB images taken at different times. The proposed numerical approach performs registration to compare the same facial palsy patients at different times. We scale landmarks by performing scale matching before global registration. After scale matching, coarse registration is performed with global registration. Point-to-plane ICP is performed using the transformation matrix obtained from global registration as the initial matrix. After registration, the distance symmetry, angular symmetry, and amount of landmark movement are calculated for the left and right sides of the face. The degree of facial palsy at a certain point in time can be approached numerically and can be compared with the degree of palsy at other times. For the same facial expressions, the degree of facial palsy at different times can be measured through distance and angle symmetry. For different facial expressions, the simultaneous degree of facial palsy in the left and right sides can be compared through the amount of landmark movement. Through experiments, the proposed method was tested using the facial palsy patient database at different times. The experiments involved clinicians and confirmed that using the proposed numerical approach can help assess the progression of facial palsy.

## 1. Introduction

Facial palsy [1,2,3] refers to paralysis of the face due to a problem in the functionality of the facial nerve that moves the muscles of the face. If the initial treatment [4,5,6] for facial palsy is not done properly, there could be serious sequelae. Additionally, it can cause external discomfort, psychological anxiety, and depression. Therefore, an accurate diagnosis of facial palsy is necessary, and it is important to accurately determine the degree of facial palsy progression. Although there is a visual method of diagnosis conducted by clinicians, this is subjective. Hence, a quantitative value that can be helpful for evaluation is required.

In recent years, quantitative evaluation of facial palsy has been studied in several ways. Optical markers [7,8,9] have been used to measure the degree of facial palsy by attaching a marker to the face. The marker is an active or passive optical marker or a gyro marker. Additionally, scanning using a laser [10,11,12] has been used to analyze facial palsy by 3D-scanning the face with an optical scanner. Although these methods very accurately determine facial features, they require additional equipment and a constrained environment; moreover, the patient feels uncomfortable when using them. RGB-D information [13,14,15,16] has also been used to extract the landmarks of the face by capturing the face using a depth camera. Although the RGB-D information method is relatively accurate, a depth camera is required. In addition, for accurate measurements, there must be a certain distance between the camera and the user. Therefore, evaluation through RGB imaging [17,18] with less restrictions on equipment and environment is an active field of study. However, the RGB imaging method uses only 68 facial landmarks, which limits the technique in terms of accuracy for muscles such as cheeks; moreover, the evaluation method is a score game for recovery or it detects whether or not facial palsy is present. Therefore, it is not possible to accurately determine the degree of facial palsy at different times.

This paper used a mediapipe [19,20,21] to extract 478 3D facial landmarks from RGB images. After extracting 3D facial landmarks, we propose a method of matching these landmarks with other facial expressions or images taken at different times. In addition, we propose three numerical approaches to measuring the progression of facial palsy after data registration. With the proposed method, the symmetry of the face can be measured and the amount of movement of the facial landmark can be obtained according to the change in this expression. Data from four patients with facial palsy were used for the evaluation of this experiment, and the consistency of the results compared to the clinician’s evaluation was confirmed. The remainder of this paper is organized as follows. Section 2 briefly reviews previous related studies. The proposed registration method and numerical approach are presented in Section 3. The experimental results for the proposed methods are reported in Section 4. Finally, we discuss and conclude the paper in Section 5.

## 2. Related Work

### 2.1. 3D Facial Landmark Localization

Three-dimensional facial landmark localization is a method of determining the location of a 3D facial landmark in a single image. In previous studies, methods for detecting 3D facial landmarks in 2D images have typically been classified into two types. A representative method [22,23,24,25] of 3D facial landmark localization using two stages involves extracting a 2D heatmap for facial landmark from a 2D image, and then expanding it to a 3D image. Although it has contributed to the study of extracting 3D landmarks from 2D, it requires a large amount of computation; hence, the one-stage methods were studied. The one-stage method estimates 3D facial landmarks without going through 2D heatmaps. Refs. [26,27,28] explore a one-stage method using scanning data and show a faster operation than the two-stage method, but it has the disadvantage of requiring scanning devices. Another one-stage method [29,30,31,32] estimates 3D facial landmarks from a single image without requiring 2D heatmaps.

However, these methods estimate a limited number of 68 landmarks. More landmark information is needed to accurately measure the progression of facial palsy. In a recent study, Kartynnik et al. [20] proposed a 3D facial landmark detector for estimating 3D mesh representations of human faces for AR apps. It uses Blazeface [33] to detect faces and extract 3D landmarks for the detected faces. After estimating 3D mesh vertices, it treats each vertex as a landmark. It operates in real time and extracts 468 facial landmarks. In Grishchenko et al. [19], in addition to these 468 landmarks, the eyes and mouth are further refined, and 10 iris landmarks are detected. In mediapipe [21], these data are provided in a modularized library; hence, it is easy to develop applications using AI functions from this library.

### 2.2. Assessment of Facial Palsy

Several studies have been conducted for the quantitative evaluation of facial palsy. Refs. [34,35,36,37,38] introduced a 3D-surface-based measurement using a 3D scanner to measure face symmetry, and [14,15,16,39] developed a 3D motion capture system using an RGB-D camera. However, these methods are still equipment-dependent. Ref. [40] developed a 3D VAS system to track dense 3D geometry, but had to manually annotate it frame by frame. For studies using machine learning, the existence of facial palsy is detected through a support vector machine [41,42,43,44] or by using a classifier [18,45]. Ngo et al. [46] evaluated the degree of facial palsy by estimating 3D facial landmarks using multiple RGB cameras. This method utilizes 3D angles and distance information but is independent of each axis. In addition, there are still limitations as multiple cameras are required, and feature information is limited because only 68 face landmarks are used. Liu et al. [47] graded degrees of facial palsy and trained the RF model using 2D facial landmarks as a feature. These gradings can be subjective and are not suitable for measuring the progression of facial paralysis in each patient. Barrios et al. [17] proposed a quantitative evaluation of facial palsy using the action unit [48] (AU) to determine the extent to which the left and right sides express the individual AUs. It is worthwhile to measure the left and right sides of the face separately, but this cannot capture the use of each muscle and does not use 3D information. Hence, our study used 3D information and captured the use of each muscle.

## 3. Proposed Methods

**Figure 1:**
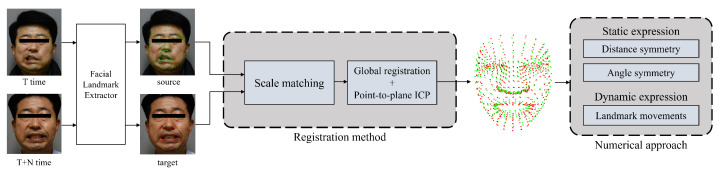
Overall framework of our proposed method. Iterative closet point (ICP) is an algorithm that finds a correlation using the closest point of each data, and moves and rotates the data to register it.

In this section, we propose a numerical approach method to evaluate facial palsy. The overall framework of the proposed numerical approach for facial palsy is shown in Figure 1. The input image used is assumed to be a frontal face. We used mediapipe [21] to extract 478 landmarks from RGB images, consisting of 468 facial landmarks and 10 eye landmarks. Landmarks from different times cannot be compared because their scale, rotation, and movement are different. Therefore, we aligned the coordinate system through registration. We propose a numerical approach to evaluate the degree of facial palsy after registration. The symmetry value of facial palsy was obtained using the distance symmetry and angle symmetry within one image. In addition, the amount of movement of the landmark of the same index extracted from two images was measured, and the amount of movement of the landmark corresponding to each side of the face was compared.

### 3.1. Registration Method

**Figure 2:**
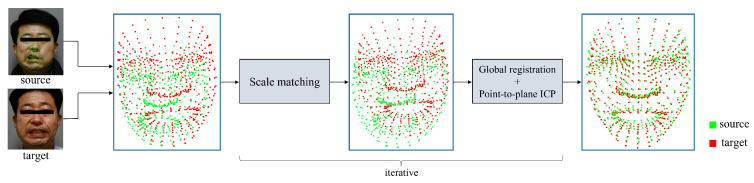
Registration flow of our proposed registration method. This method proceeds with registration after scale matching.

We propose a method for matching 3D facial landmarks extracted from RGB images at different times and with different facial expressions. For matching, it is necessary to consider that the corresponding landmark points are in the same area, but the location varies depending on the degree of facial palsy and facial expression. Euler transformation [49] has been used to register several landmarks; however, this is not applicable here because the facial muscles are mutually related. Additionally, the muscles that move depending on the facial expression are significantly different. In this method, it is therefore necessary to set landmarks to match each facial expression. Another representative registration method is the use of the iterative closest point (ICP) [50,51]. However, this method of registration for a numerical approach to facial palsy may not be suitable. For ICP, an initial transformation matrix is required. This can be matched to several facial landmarks; however, similar to the Euler transform, it is not rational to choose a fixed landmark for a particular facial expression. Therefore, we propose a registration method that does not fix landmarks. The proposed registration method is shown in Figure 2. We performed point-to-plane ICP after implementing global registration that does not require an initial transformation matrix. Let landmarks extracted from images taken at time T be the source landmarks, and landmarks extracted from images at time T+N be the target landmarks. Source landmarks and target landmarks are as follows:

(1) $$\begin{array}{c} \mathrm{source}=\left\{\left(s_{0,x},s_{0,y},s_{0,z}\right),\cdots ,\left(s_{i,x},s_{i,y},s_{i,z}\right),\cdots ,\left(s_{n-1,x},s_{n-1,y},s_{n-1,z}\right)\right\},\\ \mathrm{target}=\left\{\left(t_{0,x},t_{0,y},t_{0,z}\right),\cdots ,\left(t_{i,x},t_{i,y},t_{i,z}\right),\cdots ,\left(t_{n-1,x},t_{n-1,y},t_{n-1,z}\right)\right\} \end{array}$$

where *n* is the number of facial landmarks, which is 478 in this paper, i∈{0,n−1}.

The proposed matching algorithm is as follows:

1. Because global registration does not involve scale alignment, scale matching is performed before global registration. We can acquire the scale factor through the *i*-th landmark of the source and target landmarks. All target landmarks are then scaled using the scale factor to perform scale matching. The formula to calculate the scale factor is given as follows:
  (2) $$scale_{i}=\frac{\sqrt{{\left(s_{i,x}-O_{x}\right)}^{2}+{\left(s_{i,y}-O_{y}\right)}^{2}+{\left(s_{i,z}-O_{z}\right)}^{2}}}{\sqrt{{\left(t_{i,x}-O_{x}\right)}^{2}+{\left(t_{i,y}-O_{y}\right)}^{2}+{\left(t_{i,z}-O_{z}\right)}^{2}}}=\frac{\sqrt{s_{i,x}^{2}+s_{i,y}^{2}+s_{i,z}^{2}}}{\sqrt{t_{i,x}^{2}+t_{i,y}^{2}+t_{i,z}^{2}}}$$
  where $O_{i}=\left(O_{x},O_{y},O_{z}\right)$ is the origin of landmark coordinate. In this paper, the origin of the landmark coordinate is (0, 0, 0).
2. After scale matching, global registration that does not require an initial transformation matrix is performed for all 3D facial landmarks to perform coarse registration. Subsequently, for fine registration, the transformation matrix resulting from global registration is set as the initial transformation matrix and point-to-plane ICP [50] was performed. Point-to-plane ICP is a method to find a transformation matrix that minimizes the distance between the source landmarks and the plane of the normal vectors of target landmarks.
3. Through *n* iteration of steps 1 and 2, we obtain the total matrix $T=\left\{T_{0},\cdots ,T_{n-1}\right\}$. $T_{i}$ is the transformation matrix registered as the scale factor of the *i*-th landmark. Each $T_{i}$ is composed of a 4 × 4 matrix that represents the transformation matrix for a 3D landmark in a homogeneous coordinate method. We select the transformation matrix with the smallest inlier RMSE among *T* as the final transformation matrix, i.e., $T_{f}=T_{min}$. The inlier RMSE is defined as follows:
  (3) $$RMSE_{inlier}=\sqrt{\frac{\left(e_{0}^{2}+\cdots +e_{i}^{2}+\cdots +e_{n-1}^{2}\right)}{n}}$$
  where $e_{i}$ is calculated as the L2 distance between the *i*-th landmarks and *i*-th landmarks after converting the source landmarks using the matrix $T_{f}$, and *n* is the number of facial landmarks.
  After registration through the final transformation matrix, numerical approach is performed.

### 3.2. Numerical Approach

**Figure 3:**
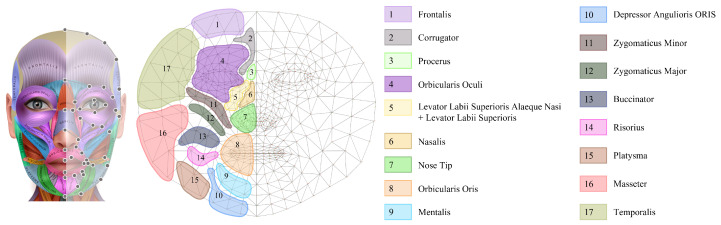
Facial muscles [52] (**left**), grouped from facial landmarks (**middle**) and indexes and muscle names (**right**). Adapted with permission from [52]. 2022, reineg.

Once the source landmarks and target landmarks have been registered, a numerical approach to evaluate the degree of facial palsy is possible. Having each value of the 478 3D facial landmarks is useful for computation; however, many values may be inefficient in helping clinicians. As depicted in Figure 3, we grouped 3D facial landmarks according to facial muscles, resulting in 17 groups, where the name of each muscle and its location are shown. No. 5 is the Levator Labii Superioris and Levator Labii Superioris Alaeque Nasi; No. 7 does not represent a muscle, but the nose tip, which is a useful area for numerical analysis because this bends under the influence of palsy of the surrounding muscles. Table 1 and Table 2 show the 478 and 68 facial landmarks included within each muscle group. We can see that using 478 landmarks provides more information about the facial muscles. A numerical approach for each muscle is then obtained by averaging the values of that muscle region.

**Figure 4:**
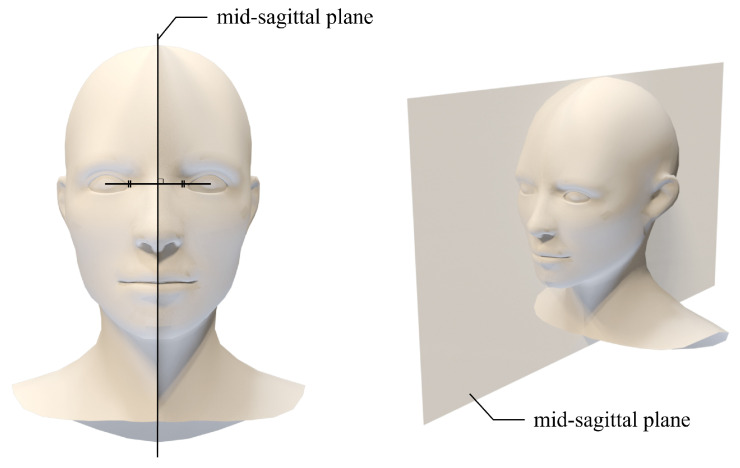
Facial midsagittal plane. The vertical bisector of the line connecting the irises is the midsagittal line, which is expanded to a 3D image to define the midsagittal plane.

In this paper, we propose three numerical approaches, including distance and angle symmetry, to analyze the same expression and amount of landmark movement obtained from different facial expressions after registration. First, in order to measure the symmetry of the face, the midsagittal plane is required. The midsagittal plane [53,54,55,56] is defined as the midline of the perpendicular bisector of the line connecting each iris. We extended this midsagittal plane of the face to 3D, as shown in Figure 4. The vector connecting each iris landmark is defined as the normal vector, and the plane passing through the midpoint of the iris landmark is defined as a facial midsagittal plane.

#### 3.2.1. Distance Symmetry

**Figure 5:**
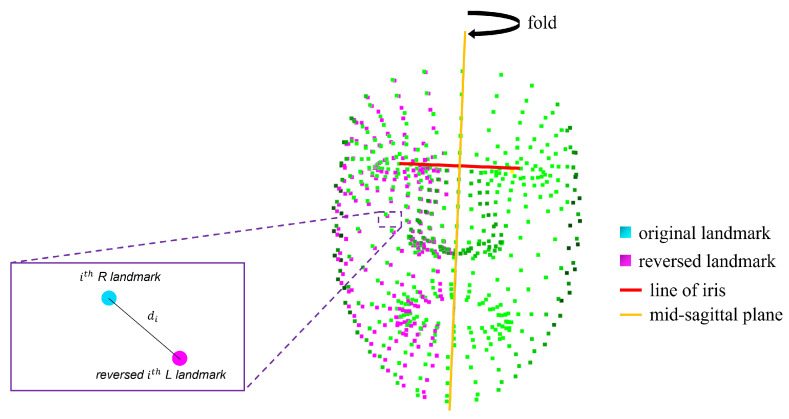
Distance symmetry. The distance from the inverted landmark (pink) after inverting the landmark on the left side of the face through the midsagittal plane.

The method to obtain the distance symmetry is shown in Figure 5. Distance symmetry is obtained by inverting the left side of the face of a person through the facial midsagittal plane. As the distance symmetry gets smaller, this implies a more perfect symmetry. Distance symmetry is defined as follows:

(4) $$d_{i}=\sqrt[{}]{{\left(p_{i,x}^{R}-p_{i,x}^{rL}\right)}^{2}+{\left(p_{i,y}^{R}-p_{i,y}^{rL}\right)}^{2}+{\left(p_{i,z}^{R}-p_{i,z}^{rL}\right)}^{2}}$$

where $p_{i}^{R}$ is the landmark of the right side of the face, and $p_{i}^{rL}$ is the landmark of the left side of the face landmark as inverted through the facial midsagittal plane.

#### 3.2.2. Angle Symmetry

**Figure 6:**
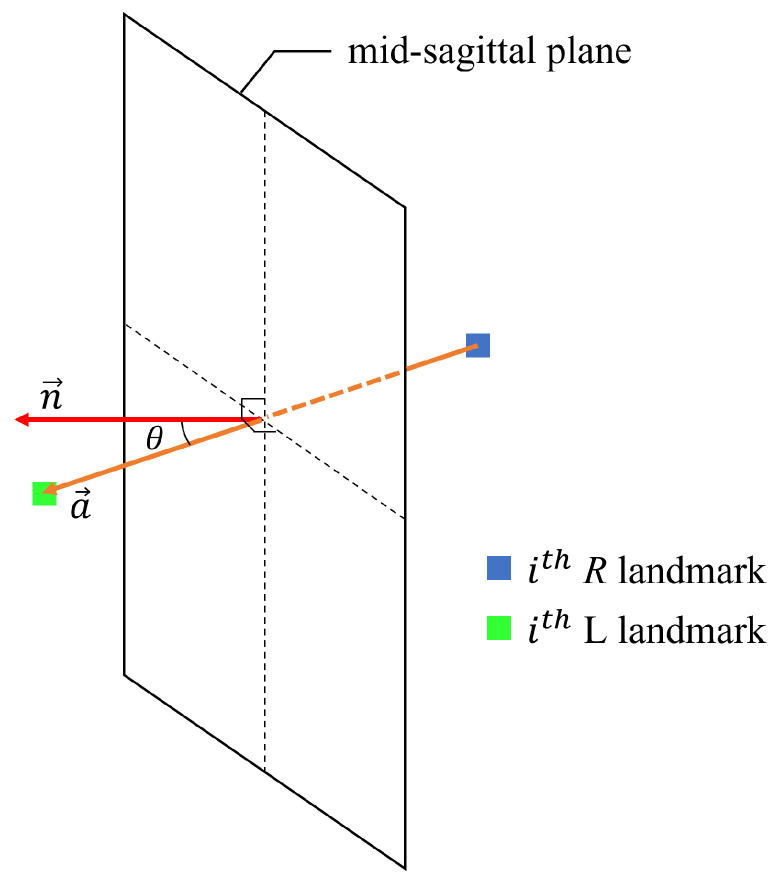
Angle symmetry through the cosine similarity of the normal vector of the midsagittal plane and the landmark pair vector.

As distance symmetry considers only symmetry with respect to distance, a numerical approach considering angle is additionally required. If the normal vector for the midsagittal plane and the vector of the left and right facial landmark pairs are the same, it means the face has left–right symmetry. A depiction of angle symmetry is shown in Figure 6. $\vec{n}$ is the normal vector of midsagittal plane, and $\vec{a}$ is the *i*-th facial landmark pair vector. The *i*-th facial landmark pair vector is the vector of the left and right pair landmarks, as shown in Equation (5):

(5) $$\vec{a}:i^{th}\;facial\;landmark\;pair\;vector=\left(\left(p_{i,x}^{R}-p_{i,x}^{L}\right),\left(p_{i,y}^{R}-p_{i,y}^{L}\right),\left(p_{i,z}^{R}-p_{i,z}^{L}\right)\right)$$

where $p_{i}$ is a pair of the *i*-th landmark, $p^{R}$ is a landmark on the right side of the face, and $p^{L}$ is a landmark on the left side of the face. Angle symmetry is defined in Equation (6). Angle symmetry uses cosine similarity. A value of 0 means perfect asymmetry, and a value of 1 means perfect symmetry.

(6) $$\mathrm{angle}\;\mathrm{symmetry}=cos\left(\theta \right)=\frac{\vec{n}\cdot \vec{a}}{∥\vec{n}∥∥\vec{a}∥}=\frac{\sum_{i=1}^{n}{\vec{n}}_{i}\times {\vec{a}}_{i}}{\sqrt{\sum_{i=1}^{n}{\left({\vec{n}}_{i}\right)}^{2}}\times \sqrt{\sum_{i=1}^{n}{\left({\vec{a}}_{i}\right)}^{2}}}$$

#### 3.2.3. Landmark Movement Amounts

**Figure 7:**
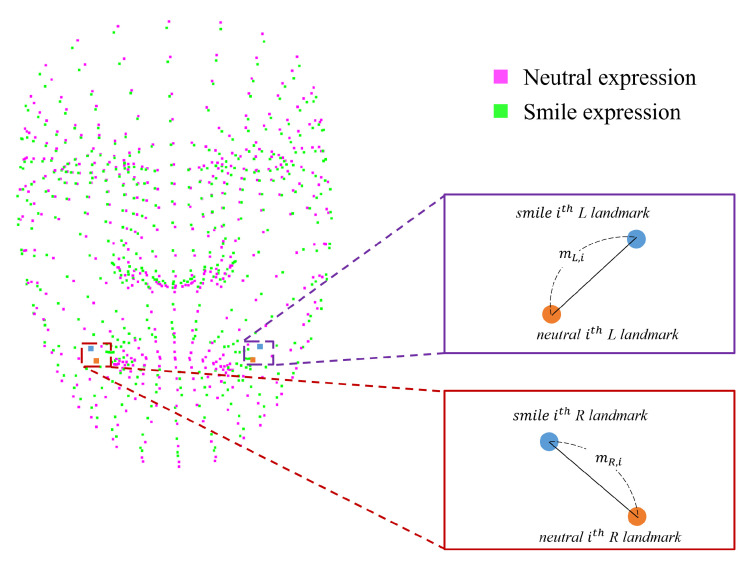
Amount of landmark movement determined through the distance of the landmarks corresponding to the left and right side of the face in the neutral and smile expression.

By simultaneously capturing neutral and smile expressions, the amount of movement of each pair of landmarks on each side of the face can be obtained. An explanation of the number of landmark movements is shown in Figure 7, in which it is possible to confirm how uniformly the left and right landmarks at the same location move in response to a change in facial expression. In addition, the amount of movement at other times and the degree of improvement in symmetry can be compared. After registration of the neutral and smile expressions, the amount of movement in the left and right landmarks corresponding to the *i*-th landmark are obtained using Equation (7). $s_{i}$ is the *i*-th landmark in the smile expression, and $n_{i}$ is the *i*-th landmark in the neutral expression.

(7) $$\begin{array}{c} i^{th}\;Left\;movementamount:m_{L,i}=\sqrt[{}]{{\left(s_{i,x}^{L}-n_{i,x}^{L}\right)}^{2}+{\left(s_{i,y}^{L}-n_{i,y}^{L}\right)}^{2}+{\left(s_{i,z}^{L}-n_{i,z}^{L}\right)}^{2}}\\ i^{th}\;Right\;movementamount:m_{R,i}=\sqrt[{}]{{\left(s_{i,x}^{R}-n_{i,x}^{R}\right)}^{2}+{\left(s_{i,y}^{R}-n_{i,y}^{R}\right)}^{2}+{\left(s_{i,z}^{R}-n_{i,z}^{R}\right)}^{2}} \end{array}$$

Therefore, the *i*-th landmark movement amount is calculated by Equation (8). A score of 0 landmark movements means perfect symmetry.

(8) $$i^{th}\;landmark\;movement=\left|i^{th}\;Left\;movements-i^{th}\;Right\;movements\right|$$

## 4. Experiments

In this section, we present the experiments conducted within this study. First, we introduce the data utilized and compare the proposed registration method with other registration methods. Next, we describe the results of applying the three numerical approach methods to facial palsy. Five clinicians participated in the experiments. The clinicians checked whether it could be helpful in the clinical evaluation of patients with facial palsy.

### 4.1. Experimental Data

**Figure 8:**
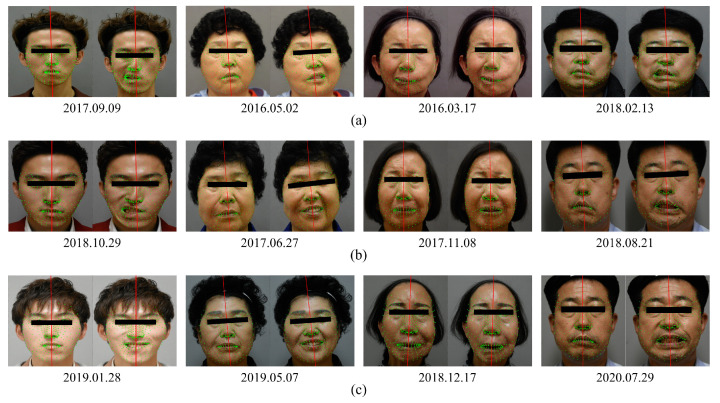
Yearly patient images. (**a**) Year 1. (**b**) Year 2. (**c**) Year 3. The green points present the extracted 3D facial landmarks, and the red horizontal line represents the line connecting the irises. The red vertical line is the midsagittal plane, which is the vertical bisector of the red horizontal line. The images, from left to right, represent Patient 1 to Patient 4.

All experiments were conducted with the consent of the patients. The dates corresponding to the first to third years of images taken of each patient are shown in Table 3. The images of the patients used in the experiment are shown in Figure 8. All RGB images were taken with a regular smartphone and webcam; a frontal face was assumed. Four patients with facial palsy were involved in this study. Images of neutral and smile expressions of each patient taken at three different times were used.

### 4.2. Experimental Results

After extracting 478 3D facial landmarks using mediapipe for patient data, we used open3D library [57], an open-source library compatible with Python. The open3D library supports the development of software that handles 3D data.

#### 4.2.1. Registration Results

All registrations used within the experiment were applied after the proposed scale matching method. After registration, the inlier RMSE was compared. As we assumed a frontal face, the initial transformation matrix of point-to-point ICP [58] and point-to-plane ICP [50] was configured using Equation (9), where *s* is the source landmark, *t* is the target landmark, and the translation matrix is set as the centroid of the source and target. *N* is the number of facial landmarks.

(9) $$T_{initial}=\left[R=I_{3}|T\right]=\left[\begin{array}{cccc} 1 & 0 & 0 & \frac{\sum (s_{x}-t_{x})}{N}\\ 0 & 1 & 0 & \frac{\sum (s_{y}-t_{y})}{N}\\ 0 & 0 & 1 & \frac{\sum (s_{z}-t_{z})}{N}\\ 0 & 0 & 0 & 1 \end{array}\right]$$

**Figure 9:**
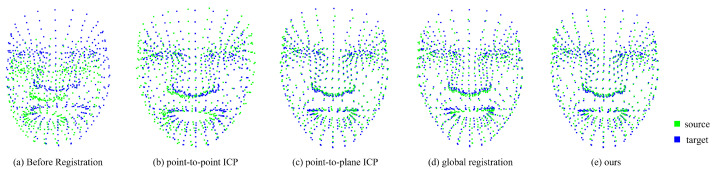
Registration comparison image in static expression.

**Figure 10:**
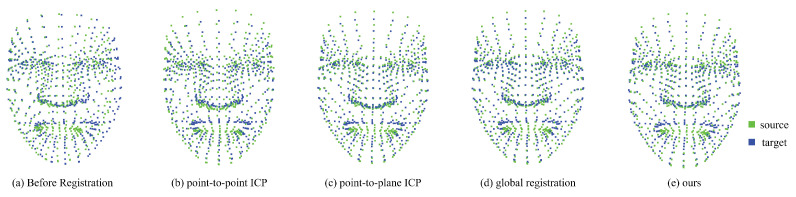
Registration comparison image in dynamic expression.

- Experiments by year
  Table 4 shows the inlier RMSE results when several matching methods were applied. Three-dimensional facial landmarks extracted from smile expression images at different times were used. We compared the inlier RMSE by registration of the Year 1-Year 2 images and Year 1-Year 3 images of each patient. All of our proposed methods had minimal inlier RMSE except for Year 1-Year 3 of Patient 2. In the case of global registration, the RMSE was similar to that of other registrations, because there was no initial transformation matrix. However, Year 1-Year 3 of Patient 2 had the smallest RMSE in the point-to-plane ICP, demonstrating that our method, without requiring an initial transformation matrix, is superior to the other methods. Figure 9 shows an example of the visualized result for Year 1-Year 3 of Patient 4.
- Experiments by expression
  Table 5 shows the RMSE when examining the neutral and smile expressions of each patient in the same year. Similar to the yearly experiments, global registration has a large RMSE compared to other registrations. Our method achieved more optimal registration, despite point-to-point ICP and point-to-plane ICP requiring an initial transformation matrix. A visualization of the results are shown in Figure 10 for the example of Year 1-Year 3 of patient 4.

#### 4.2.2. Distance and Angle Symmetry Results

Here, we describe the experimental results when using the method of measuring symmetry in the static expression. The smiling expression was examined, which is a representative expression used in the diagnosis of facial palsy. Four, six, seven, and eight facial muscle areas were used in the experiment.

- Distance Symmetry
  We obtained the distance symmetry by inverting the left side of the face through the facial midsagittal plane obtained from the distance between the irises. Table 6 shows the distance symmetry results for the four patients regarding four facial muscles. For intuitive observation, the results have been rounded to *100 and to the sixth decimal place. Distance symmetry has a positive value, so 0 means perfect symmetry. We also confirmed the agreement between the results and diagnosis of clinicians. In addition, it was confirmed that the distance symmetry of the patients became closer to perfect symmetry as time passed. Regarding the nose tip, Patient 1 had worse distance symmetry in Year 2 than in Year 1, which was consistent with the clinician’s evaluation.
- Angle Symmetry
  Angle symmetry is determined by the cosine similarity of the facial midsagittal plane and the landmark pair vector. Table 7 shows the results of the angle symmetry analysis. Similar to the distance symmetry, these results have been rounded to *100 and the sixth decimal place for intuitive observation. For distance symmetry, 100 means perfect symmetry; the smaller this value is, the more asymmetrical the face is. The cosine similarity metric is 0 when both vectors are perpendicular. As the normal vector of the facial midsagittal plane and the landmark pair vector are generally close to parallel, the angle symmetry has a value of 90 or more. As we used a smile expression in the experiment, the change in Orbicularis Oris was the greatest. We confirmed that the Orbicularis Oris was close to angular symmetry for the four patients.

#### 4.2.3. Landmark Movements Results

Landmark Movements
  This experiment involved a method of measuring symmetry in dynamic expressions. We simultaneously examined the neutral expressions and smile expressions. The closer the result was to 0, the more symmetric the expressions were, and the larger the value, the more asymmetric it was. Through experiments, we compared the balance of the movement between the left and right facial landmarks of the patient. Table 8 shows the difference in the amount of landmark movement corresponding to the left and right sides of the face for neutral and smile expressions. The Orbicularis Oris of Patient 4 was 9.501 in Year 1, but decreased to 5.285 in Year 2, and 1.391 in Year 3, resulting in a smaller amount of landmark movement. This showed that the degree of facial palsy had improved.

## 5. Conclusions and Discussion

In this study, we proposed three numerical approaches after registration for diagnosing the progression of facial palsy in patients. As RGB images have only 2D information, we attempted to obtain more information by extending this to a 3D image. To compare images at different times and with different facial expressions, registration was performed through scaling matching, global registration, and point-to-plane ICP. After registration, distance symmetry and angle symmetry, which can numerically evaluate the symmetry in the static expression, can be obtained. Symmetry in the dynamic expression was approached numerically using the amount of landmark movement. However, AI-based 3D facial landmark detection trained based on facial expressions of people without facial palsy diagnoses may cause errors when used for patients with facial palsy. In addition, due to personal information privacy problems and data limitations, we proceeded with limited expressions. We make the following contributions: First, a degree of improvement in facial palsy could be obtained numerically without sensors or depth cameras. Second, we proposed a numerical approach to measure the degree of facial palsy over time. Third, the degree of facial palsy on the left and right sides of the face could be obtained numerically according to the amount of landmark movement over time. Fourth, it is possible to accurately compare the location of muscles or landmarks at different times. It is expected that the proposed numerical approach to measuring facial palsy will help clinicians evaluate facial palsy. Future work will build and experiment with datasets with various expressions for facial palsy rehabilitation and various angles.

## Figures and Tables

**Table 1:** The 478 facial landmarks in the defined facial muscle groups.

Muscle Index and NAME	# of Landmarks	Muscle Index and Name	# of Landmarks
1. Frontalis	6	10. Depressor Anguli Oris	8
2. Corrugator	1	11. Zygomaticus Minor	6
3. Procerus	1	12. Zygomaticus Major	3
4. Orbicularis Oculi	59	13. Buccinator	4
5. Levator Labii Superioris (+ Alaeque Nasi)	10	14. Risorius	3
6. Nasalis	6	15. Platysma	5
7. Nose Tip	32	16. Masseter	9
8. Orbicularis Oris	44	17. Temporalis	16
9. Mentalis	6		

**Table 2:** The 68 facial landmarks in the defined facial muscle groups.

Muscle Index and Name	# of Landmarks	Muscle Index and Name	# of Landmarks
1. Frontalis	0	10. Depressor Anguli Oris	2
2. Corrugator	0	11. Zygomaticus Minor	0
3. Procerus	2	12. Zygomaticus Major	0
4. Orbicularis Oculi	6	13. Buccinator	0
5. Levator Labii Superioris (+ Alaeque Nasi)	0	14. Risorius	0
6. Nasalis	2	15. Platysma	2
7. Nose Tip	3	16. Masseter	4
8. Orbicularis Oris	12	17. Temporalis	1
9. Mentalis	0		

**Table 3:** Dates of images taken for each patient.

Patient	Year 1	Year 2	Year 3
Patient 1	9 September 2017	29 October 2018	28 January 2019
Patient 2	2 May 2016	27 June 2017	7 May 2019
Patient 3	17 March 2016	8 November 2017	17 December 2018
Patient 4	13 February 2018	21 August 2018	29 July 2020

**Table 4:** Registration comparison in static expression.

	Patient 1		Patient 2		Patient 3		Patient 4	
Registration Method	Year 1-Year 2	Year 1-Year 3	Year 1-Year 2	Year 1-Year 3	Year 1-Year 2	Year 1-Year 3	Year 1-Year 2	Year 1-Year 3
Before Registration	$7.99\times 10^{-2}$	$5.32\times 10^{-2}$	$5.81\times 10^{-2}$	$5.62\times 10^{-2}$	$5.63\times 10^{-2}$	$6.45\times 10^{-2}$	$9.15\times 10^{-2}$	$10.73\times 10^{-2}$
Point-to-point ICP	$1.41\times 10^{-2}$	$1.93\times 10^{-2}$	$2.15\times 10^{-2}$	$2.12\times 10^{-2}$	$1.45\times 10^{-2}$	$1.04\times 10^{-2}$	$1.99\times 10^{-2}$	$2.47\times 10^{-2}$
Point-to-plane ICP	$1.65\times 10^{-2}$	$2.23\times 10^{-2}$	$1.57\times 10^{-2}$	$1.31\times 10^{-2}$	$12.58\times 10^{-2}$	$1\times 10^{-2}$	$1.87\times 10^{-2}$	$1.67\times 10^{-2}$
Global Registration	$1.91\times 10^{-2}$	$2.5\times 10^{-2}$	$2.11\times 10^{-2}$	$1.88\times 10^{-2}$	$1.86\times 10^{-2}$	$1.38\times 10^{-2}$	$2.34\times 10^{-2}$	$2.1\times 10^{-2}$
Ours	$1.27\times 10^{-2}$	$1.61\times 10^{-2}$	$1.53\times 10^{-2}$	$1.33\times 10^{-2}$	$1.34\times 10^{-2}$	$0.91\times 10^{-2}$	$1.73\times 10^{-2}$	$1.64\times 10^{-2}$

**Table 5:** Registration comparison for dynamic expression (neutral-smile).

	Patient 1			Patient 2			Patient 3			Patient 4		
Registration Method	Year 1	Year 2	Year 3	Year 1	Year 2	Year 3	Year 1	Year 2	Year 3	Year 1	Year 2	Year 3
Before Registration	$6.49\times 10^{-2}$	$3.52\times 10^{-2}$	$3\times 10^{-2}$	$2.48\times 10^{-2}$	$3.22\times 10^{-2}$	$2.8\times 10^{-2}$	$2.03\times 10^{-2}$	$2.88\times 10^{-2}$	$1.71\times 10^{-2}$	$2.38\times 10^{-2}$	$2.2\times 10^{-2}$	$3.93\times 10^{-2}$
Point-to-point ICP	$1.47\times 10^{-2}$	$1.42\times 10^{-2}$	$1.63\times 10^{-2}$	$2.01\times 10^{-2}$	$1.14\times 10^{-2}$	$1.12\times 10^{-2}$	$1.07\times 10^{-2}$	$0.75\times 10^{-2}$	$0.78\times 10^{-2}$	$1.17\times 10^{-2}$	$1.39\times 10^{-2}$	$2.04\times 10^{-2}$
Point-to-plane ICP	$1.47\times 10^{-2}$	$1.42\times 10^{-2}$	$1.62\times 10^{-2}$	$1.14\times 10^{-2}$	$1.14\times 10^{-2}$	$1.11\times 10^{-2}$	$7.66\times 10^{-2}$	$0.64\times 10^{-2}$	$0.78\times 10^{-2}$	$1.17\times 10^{-2}$	$8.7\times 10^{-2}$	$1.72\times 10^{-2}$
Global Registration	$2.12\times 10^{-2}$	$1.62\times 10^{-2}$	$1.8\times 10^{-2}$	$1.73\times 10^{-2}$	$1.54\times 10^{-2}$	$1.55\times 10^{-2}$	$1.79\times 10^{-2}$	$1.18\times 10^{-2}$	$1.43\times 10^{-2}$	$1.57\times 10^{-2}$	$1.68\times 10^{-2}$	$2.16\times 10^{-2}$
Ours	$1.42\times 10^{-2}$	$1.27\times 10^{-2}$	$1.42\times 10^{-2}$	$1.13\times 10^{-2}$	$1.05\times 10^{-2}$	$1.09\times 10^{-2}$	$1.04\times 10^{-2}$	$0.64\times 10^{-2}$	$0.74\times 10^{-2}$	$0.96\times 10^{-2}$	$1.25\times 10^{-2}$	$1.7\times 10^{-2}$

**Table 6:** Distance symmetry results for each facial muscle of patients by year in smile expression.

Facial Muscle Index	Name	Year 1	Year 2	Year 3
4. Orbicularis Oculi	Patient 1	7.5921	0.73673	0.51192
	Patient 2	1.48942	1.18371	0.38719
	Patient 3	1.29508	2.04998	1.06607
	Patient 4	0.92826	0.67437	0.8238
6. Nasalis	Patient 1	2.22854	2.86766	1.13281
	Patient 2	0.81672	0.2776	0.22651
	Patient 3	1.19751	0.87822	0.1149
	Patient 4	1.90695	0.17484	0.21075
7. Nose Tip	Patient 1	3.44382	4.10762	0.907845
	Patient 2	1.13858	0.68551	0.36036
	Patient 3	2.08707	1.92098	0.18086
	Patient 4	2.74056	0.48394	0.64874
8. Orbicularis Oris	Patient 1	10.23885	9.4951	0.47006
	Patient 2	6.07195	1.85901	1.00492
	Patient 3	8.36119	2.69559	0.40773
	Patient 4	7.86863	1.36783	1.72486

**Table 7:** Angle symmetry results for each facial muscle of patients by year in smile expression.

Facial Muscle Index	Name	Year 1	Year 2	Year 3
4. Orbicularis Oculi	Patient 1	99.9796	99.98498	99.98784
	Patient 2	99.92574	99.98562	99.99131
	Patient 3	99.93721	99.86339	99.98162
	Patient 4	99.86334	99.96863	99.96985
6. Nasalis	Patient 1	99.9505	99.97361	99.99662
	Patient 2	99.98091	99.99106	99.99449
	Patient 3	98.82472	99.9508	99.996
	Patient 4	99.9736	99.99543	99.98499
7. Nose Tip	Patient 1	98.89051	98.94509	99.99477
	Patient 2	98.94933	99.9819	99.9842
	Patient 3	99.80846	99.93403	99.99433
	Patient 4	99.94095	99.97020	99.98882
8. Orbicularis Oris	Patient 1	98.75177	99.79075	99.98268
	Patient 2	99.6784	99.9741	99.97713
	Patient 3	99.44894	99.83701	99.98333
	Patient 4	99.74328	99.86987	99.96659

**Table 8:** Results for the amount of landmark movement of each facial muscle for patients by year in dynamic expression.

Facial Muscle index	Name	Year 1	Year 2	Year 3
4. Orbicularis Oculi	Patient 1	12.949	1.961	0.798
	Patient 2	10.174	7.581	1.087
	Patient 3	6.545	2.209	1.641
	Patient 4	6.407	4.373	3.707
6. Nasalis	Patient 1	2.415	0.582	0.319
	Patient 2	0.557	0.16	0.115
	Patient 3	0.759	0.392	0.138
	Patient 4	0.522	0.396	0.045
7. Nose Tip	Patient 1	2.723	2.049	0.418
	Patient 2	2.355	0.57	0.079
	Patient 3	0.444	0.368	0.186
	Patient 4	2.071	2.013	0.159
8. Orbicularis Oris	Patient 1	10.422	9.276	5.685
	Patient 2	4.812	2.098	0.59
	Patient 3	5.999	3.77	2.263
	Patient 4	9.501	5.285	1.391

## Data Availability

Not applicable.
